# Aqua­trichlorido(1-cyano­methyl-4-aza-1-azoniabicyclo­[2.2.2]octane-κ*N* ^4^)copper(II) monohydrate

**DOI:** 10.1107/S1600536812017205

**Published:** 2012-04-25

**Authors:** Qinqin Zhou, Yi Zhang

**Affiliations:** a Ordered Matter Science Research Center, Southeast University, Nanjing 211189, People’s Republic of China

## Abstract

The asymmetric unit of the title compound, [CuCl_3_(C_8_H_14_N_3_)(H_2_O)]·H_2_O, comprises a neutral complex and a mol­ecule of free water. The complex contains coordinated Cu^II^ ions, with Cu—Cl distances ranging from 2.3471 (8) to 2.4011 (8) Å, and with Cu—N and Cu—O distances of 2.0775 (19) and 2.0048 (18) Å, respectively. The resulting coordination polyhedron is a trigonal bipyramid with the Cl atoms in the equatorial plane. In the crystal, O—H⋯Cl and O—H⋯O hydrogen bonds link the mol­ecules into a three-dimensional structure.

## Related literature

For background to dielectric-ferroelectric materials, see: Fu *et al.* (2010 ▶); Zhang *et al.* (2008 ▶). The title compound was prepared in an attempt to make analogs of (dabcoH_2_)_2_Cl_3_[CuCl_3_(H_2_O)_2_]·H_2_O (Wei & Willett, 1996 ▶) and (dabcoH_2_)CuCl_4_ and Zn(dabcoH)Cl_3_ (Wei & Willett, 2001 ▶) (dabco is 1,4-diazabicyclo[2.2.2]octan). 
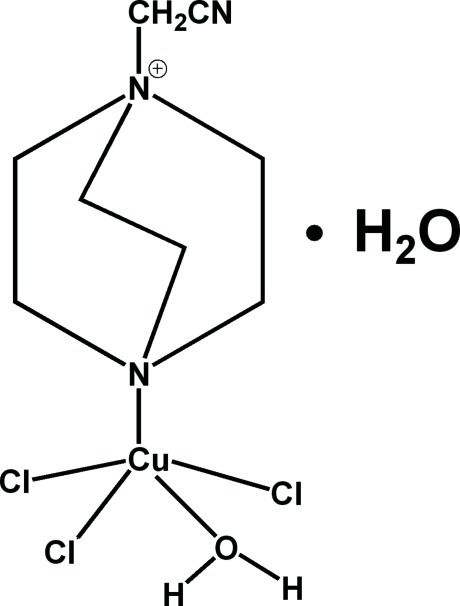


## Experimental

### 

#### Crystal data

[CuCl_3_(C_8_H_14_N_3_)(H_2_O)]·H_2_O
*M* *_r_* = 358.14
Monoclinic, 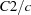

*a* = 24.301 (5) Å
*b* = 8.2794 (17) Å
*c* = 14.069 (3) Å
β = 101.69 (3)°
*V* = 2771.9 (10) Å^3^
*Z* = 8
Mo *K*α radiation
μ = 2.15 mm^−1^
*T* = 298 K
0.36 × 0.32 × 0.28 mm

#### Data collection

Rigaku SCXmini diffractometer
Absorption correction: multi-scan (*CrystalClear*; Rigaku, 2005 ▶) *T*_min_ = 0.963, *T*_max_ = 0.971
13618 measured reflections
3155 independent reflections
2881 reflections with *I* > 2σ(*I*)
*R*_int_ = 0.068

#### Refinement

*R*[*F* ^2^ > 2σ(*F* ^2^)] = 0.036
*wR*(*F* ^2^) = 0.093
*S* = 1.10
3155 reflections
170 parameters
H atoms treated by a mixture of independent and constrained refinement
Δρ_max_ = 0.62 e Å^−3^
Δρ_min_ = −0.86 e Å^−3^

### 

Data collection: *CrystalClear* (Rigaku, 2005 ▶); cell refinement: *CrystalClear*; data reduction: *CrystalClear*; program(s) used to solve structure: *SHELXS97* (Sheldrick, 2008 ▶); program(s) used to refine structure: *SHELXL97* (Sheldrick, 2008 ▶); molecular graphics: *SHELXTL* (Sheldrick, 2008 ▶); software used to prepare material for publication: *SHELXTL*.

## Supplementary Material

Crystal structure: contains datablock(s) I, global. DOI: 10.1107/S1600536812017205/fj2535sup1.cif

Structure factors: contains datablock(s) I. DOI: 10.1107/S1600536812017205/fj2535Isup2.hkl

Additional supplementary materials:  crystallographic information; 3D view; checkCIF report

## Figures and Tables

**Table 1:** Hydrogen-bond geometry (Å, °)

*D*—H⋯*A*	*D*—H	H⋯*A*	*D*⋯*A*	*D*—H⋯*A*
O2—H13⋯Cl2^i^	0.93 (4)	2.24 (4)	3.128 (2)	159 (3)
O2—H12⋯O1	0.80 (4)	1.92 (4)	2.693 (3)	160 (4)
O1—H11⋯Cl3^ii^	0.77 (5)	2.72 (5)	3.447 (3)	159 (4)
O1—H10⋯Cl3^i^	0.80 (5)	2.54 (6)	3.337 (3)	171 (5)

Symmetry codes: (i) 
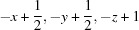
; (ii) 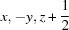.

**Fig. 1.**
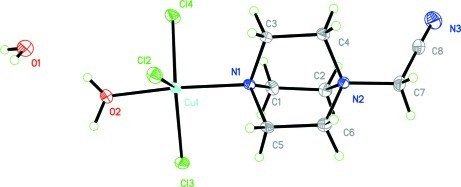
The molecular structure of the title compound, showing the atomic numbering scheme with 30% probability displacement ellipsoids.

**Fig. 2.**
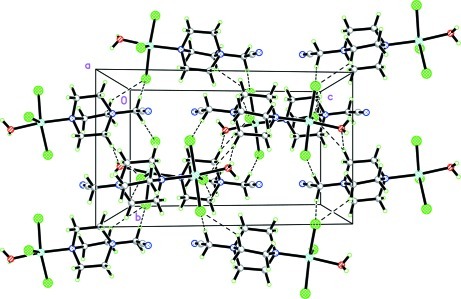
A view of the packing of the title compound, stacking along the a axis. Dashed lines indicate hydrogen bonds.

## supplementary crystallographic information

### Comment

The study of ferroelectric materials has received much attention and some materials have predominantly dielectric-ferroelectric performance (Fu *et al.*(2010); Zhang *et al.*(2008)). The title compound was prepared in an attempt to make analogs to (dabcoH_2_)_2_Cl_3_[CuCl_3_(H_2_O)_2_].H_2_O (Wei & Willett, 1996) and to (dabcoH_2_)CuCl_4_ and Zn(dabcoH)Cl_3_(Wei & Willett, 2001).

The asymmetric unit of the title compound, (dabcoCH_2_CN)[CuCl_3_(H_2_O)].H_2_O(dabco is 1,4-bicyclo[2.2.2]octane), comprises a (dabcoCH_2_CN)[CuCl_3_(H_2_O)] moleculeand a molecule of free water.The Cu(dabcoCH_2_CN)Cl_3_(H_2_O) molecule coordinated Cu^II^ ion has Cu—Cl distances ranging from 2.347 (8) to 2.401 (8) Å, a Cu—N distance of 2.078 (19) Å and a Cu—O distance of 2.005 (18) Å.There are hydrogen bonds found which are O(1)—H(10)···Cl(3), O(1)—H(11)···Cl(3), O(2)—H(13)···Cl(2), O(1)—H(12)···O(1).The hydrogen-bonded sheets link the molecules into a three-dimensional structure.

### Experimental

(dabcoCH_2_CN)Cl(10 mmol,1.68 g) were dissolved in 15 mL water,thenCuCl_2_^.^H_2_O (10 mmol, 1.70 g) in 15 ml water was added into the previous solution and the mixed solution was filtered last. After a few days a great quantity of green microcrystasls were obtained by by slow evaporation at room temperature in air.

Data collection: *CrystalClear* (Rigaku, 2005); cell refinement: *CrystalClear*; data reduction: *CrystalClear*; program(*s*) used to solve structure: *SHELXS97* (Sheldrick, 2008); program(*s*) used to refine structure: *SHELXL97* (Sheldrick, 2008); molecular graphics: *SHELXTL/PC* (Sheldrick, 2008); software used to prepare material for publication: *SHELXTL/PC*.

### Refinement

H atoms were placed in calculated positions(C—H = 0.97 Å for C*sp^3^* atoms), assigned fixed *U*_iso_ values [*U*_iso_ = 1.2*U*eq(C*sp^2^*/N) and 1.5*U*eq(C*sp^3^*)] and allowed to ride.

### Crystal data

**Table:**

[CuCl_3_(C_8_H_14_N_3_)(H_2_O)]·H_2_O	*F*(000) = 1464
*M**_r_* = 358.14	*D*_x_ = 1.716 Mg m^−^^3^
Monoclinic, *C*2/*c*	Mo *K*α radiation, λ = 0.71073 Å
Hall symbol: -C 2yc	Cell parameters from 12903 reflections
*a* = 24.301 (5) Å	θ = 3.1–27.5°
*b* = 8.2794 (17) Å	µ = 2.15 mm^−^^1^
*c* = 14.069 (3) Å	*T* = 298 K
β = 101.69 (3)°	Block, green
*V* = 2771.9 (10) Å^3^	0.36 × 0.32 × 0.28 mm
*Z* = 8	

### Data collection

**Table:**

Rigaku SCXmini diffractometer	3155 independent reflections
Radiation source: fine-focus sealed tube	2881 reflections with *I* > 2σ(*I*)
Graphite monochromator	*R*_int_ = 0.068
Detector resolution: 13.6612 pixels mm^-1^	θ_max_ = 27.5°, θ_min_ = 3.1°
ω scans	*h* = −31→31
Absorption correction: multi-scan (*CrystalClear*; Rigaku, 2005)	*k* = −10→10
*T*_min_ = 0.963, *T*_max_ = 0.971	*l* = −18→18
13618 measured reflections	

### Refinement

**Table:**

Refinement on *F*^2^	Primary atom site location: structure-invariant direct methods
Least-squares matrix: full	Secondary atom site location: difference Fourier map
*R*[*F*^2^ > 2σ(*F*^2^)] = 0.036	Hydrogen site location: inferred from neighbouring sites
*wR*(*F*^2^) = 0.093	H atoms treated by a mixture of independent and constrained refinement
*S* = 1.10	*w* = 1/[σ^2^(*F*_o_^2^) + (0.037*P*)^2^ + 2.7164*P*] where *P* = (*F*_o_^2^ + 2*F*_c_^2^)/3
3155 reflections	(Δ/σ)_max_ < 0.001
170 parameters	Δρ_max_ = 0.62 e Å^−^^3^
0 restraints	Δρ_min_ = −0.86 e Å^−^^3^

### Special details

**Table:**

Geometry. All e.s.d.'s (except the e.s.d. in the dihedral angle between two l.s. planes) are estimated using the full covariance matrix. The cell e.s.d.'s are taken into account individually in the estimation of e.s.d.'s in distances, angles and torsion angles; correlations between e.s.d.'s in cell parameters are only used when they are defined by crystal symmetry. An approximate (isotropic) treatment of cell e.s.d.'s is used for estimating e.s.d.'s involving l.s. planes.
Refinement. Refinement of *F*^2^ against ALL reflections. The weighted *R*-factor *wR* and goodness of fit *S* are based on *F*^2^, conventional *R*-factors *R* are based on *F*, with *F* set to zero for negative *F*^2^. The threshold expression of *F*^2^ > σ(*F*^2^) is used only for calculating *R*-factors(gt) *etc*. and is not relevant to the choice of reflections for refinement. *R*-factors based on *F*^2^ are statistically about twice as large as those based on *F*, and *R*- factors based on ALL data will be even larger.

### Fractional atomic coordinates and isotropic or equivalent isotropic displacement parameters (Å^2^)

**Table:**

	*x*	*y*	*z*	*U*_iso_*/*U*_eq_	
C1	0.08955 (10)	0.0927 (3)	0.17062 (17)	0.0250 (5)	
H1A	0.0550	0.0937	0.1951	0.030*	
H1B	0.1083	−0.0094	0.1887	0.030*	
C2	0.07568 (10)	0.1079 (3)	0.05965 (17)	0.0246 (5)	
H2A	0.0922	0.0182	0.0309	0.030*	
H2B	0.0353	0.1042	0.0366	0.030*	
C3	0.09449 (10)	0.3817 (3)	0.19080 (16)	0.0215 (5)	
H3A	0.1182	0.4723	0.2164	0.026*	
H3B	0.0621	0.3820	0.2212	0.026*	
C4	0.07485 (10)	0.4018 (3)	0.08053 (16)	0.0232 (5)	
H4A	0.0341	0.4002	0.0636	0.028*	
H4B	0.0877	0.5047	0.0603	0.028*	
C5	0.17741 (9)	0.2318 (3)	0.17037 (17)	0.0215 (5)	
H5A	0.1965	0.1285	0.1806	0.026*	
H5B	0.2031	0.3142	0.2019	0.026*	
C6	0.16180 (10)	0.2671 (3)	0.06133 (18)	0.0261 (5)	
H6A	0.1764	0.3718	0.0479	0.031*	
H6B	0.1782	0.1859	0.0258	0.031*	
C7	0.08423 (11)	0.2867 (3)	−0.07951 (18)	0.0300 (6)	
H7A	0.0986	0.3898	−0.0964	0.036*	
H7B	0.1023	0.2023	−0.1099	0.036*	
C8	0.02328 (12)	0.2805 (3)	−0.11707 (18)	0.0314 (6)	
Cl2	0.20113 (3)	0.44899 (7)	0.38684 (4)	0.02851 (16)	
Cl3	0.19677 (3)	−0.05276 (7)	0.34199 (4)	0.02920 (16)	
Cl4	0.06069 (3)	0.23400 (10)	0.39370 (5)	0.03668 (18)	
Cu1	0.151563 (11)	0.19653 (3)	0.364527 (19)	0.01994 (11)	
H10	0.217 (2)	0.384 (6)	0.666 (3)	0.091 (19)*	
H11	0.187 (2)	0.295 (5)	0.709 (3)	0.074 (17)*	
H12	0.1751 (17)	0.215 (5)	0.543 (3)	0.063 (13)*	
H13	0.2148 (17)	0.111 (4)	0.521 (3)	0.055 (10)*	
N1	0.12648 (8)	0.2280 (2)	0.21552 (13)	0.0166 (4)	
N2	0.09851 (8)	0.2654 (2)	0.02936 (14)	0.0197 (4)	
N3	−0.02358 (12)	0.2753 (4)	−0.14739 (19)	0.0471 (7)	
O1	0.18732 (11)	0.3383 (3)	0.66114 (18)	0.0418 (5)	
O2	0.17734 (8)	0.1430 (2)	0.50550 (13)	0.0276 (4)	

### Atomic displacement parameters (Å^2^)

**Table:**

	*U*^11^	*U*^22^	*U*^33^	*U*^12^	*U*^13^	*U*^23^
C1	0.0211 (12)	0.0239 (12)	0.0279 (12)	−0.0077 (10)	0.0001 (9)	0.0016 (9)
C2	0.0241 (12)	0.0221 (12)	0.0263 (12)	−0.0055 (10)	0.0018 (9)	−0.0047 (9)
C3	0.0207 (11)	0.0226 (12)	0.0208 (11)	0.0067 (9)	0.0030 (9)	0.0008 (8)
C4	0.0241 (12)	0.0215 (12)	0.0228 (12)	0.0048 (9)	0.0019 (9)	−0.0007 (9)
C5	0.0121 (11)	0.0287 (12)	0.0238 (12)	0.0007 (9)	0.0036 (9)	0.0016 (9)
C6	0.0133 (12)	0.0402 (14)	0.0255 (13)	−0.0021 (10)	0.0057 (9)	0.0013 (10)
C7	0.0272 (14)	0.0432 (16)	0.0190 (12)	0.0016 (11)	0.0035 (10)	−0.0018 (10)
C8	0.0329 (16)	0.0393 (15)	0.0204 (12)	0.0038 (12)	0.0013 (11)	−0.0021 (10)
Cl2	0.0248 (3)	0.0267 (3)	0.0309 (3)	−0.0035 (2)	−0.0016 (2)	−0.0039 (2)
Cl3	0.0303 (3)	0.0266 (3)	0.0309 (3)	0.0101 (2)	0.0067 (2)	0.0034 (2)
Cl4	0.0170 (3)	0.0620 (5)	0.0328 (4)	0.0085 (3)	0.0093 (3)	0.0129 (3)
Cu1	0.01484 (17)	0.02434 (18)	0.01982 (17)	0.00123 (10)	0.00157 (11)	0.00118 (10)
N1	0.0110 (9)	0.0189 (9)	0.0196 (9)	−0.0008 (7)	0.0023 (7)	0.0001 (7)
N2	0.0159 (10)	0.0259 (10)	0.0172 (9)	0.0007 (8)	0.0029 (7)	−0.0018 (7)
N3	0.0357 (16)	0.0609 (18)	0.0387 (15)	0.0060 (13)	−0.0065 (11)	−0.0060 (12)
O1	0.0392 (14)	0.0456 (13)	0.0378 (13)	0.0086 (11)	0.0013 (10)	−0.0065 (10)
O2	0.0252 (10)	0.0324 (10)	0.0229 (9)	0.0049 (8)	−0.0007 (7)	0.0000 (7)

### Geometric parameters (Å, º)

**Table:**

C1—N1	1.493 (3)	C6—N2	1.513 (3)
C1—C2	1.534 (3)	C6—H6A	0.9700
C1—H1A	0.9700	C6—H6B	0.9700
C1—H1B	0.9700	C7—C8	1.469 (4)
C2—N2	1.512 (3)	C7—N2	1.511 (3)
C2—H2A	0.9700	C7—H7A	0.9700
C2—H2B	0.9700	C7—H7B	0.9700
C3—N1	1.495 (3)	C8—N3	1.133 (4)
C3—C4	1.537 (3)	Cl2—Cu1	2.4011 (8)
C3—H3A	0.9700	Cl3—Cu1	2.3893 (7)
C3—H3B	0.9700	Cl4—Cu1	2.3471 (8)
C4—N2	1.514 (3)	Cu1—O2	2.0048 (18)
C4—H4A	0.9700	Cu1—N1	2.0775 (19)
C4—H4B	0.9700	O1—H10	0.80 (5)
C5—N1	1.502 (3)	O1—H11	0.77 (5)
C5—C6	1.532 (3)	O2—H12	0.80 (4)
C5—H5A	0.9700	O2—H13	0.93 (4)
C5—H5B	0.9700		
			
N1—C1—C2	111.02 (18)	H6A—C6—H6B	108.3
N1—C1—H1A	109.4	C8—C7—N2	111.6 (2)
C2—C1—H1A	109.4	C8—C7—H7A	109.3
N1—C1—H1B	109.4	N2—C7—H7A	109.3
C2—C1—H1B	109.4	C8—C7—H7B	109.3
H1A—C1—H1B	108.0	N2—C7—H7B	109.3
N2—C2—C1	109.86 (18)	H7A—C7—H7B	108.0
N2—C2—H2A	109.7	N3—C8—C7	179.0 (3)
C1—C2—H2A	109.7	O2—Cu1—N1	174.24 (8)
N2—C2—H2B	109.7	O2—Cu1—Cl4	88.43 (6)
C1—C2—H2B	109.7	N1—Cu1—Cl4	93.78 (6)
H2A—C2—H2B	108.2	O2—Cu1—Cl3	83.13 (6)
N1—C3—C4	111.57 (18)	N1—Cu1—Cl3	91.32 (5)
N1—C3—H3A	109.3	Cl4—Cu1—Cl3	127.71 (3)
C4—C3—H3A	109.3	O2—Cu1—Cl2	90.87 (6)
N1—C3—H3B	109.3	N1—Cu1—Cl2	93.43 (6)
C4—C3—H3B	109.3	Cl4—Cu1—Cl2	109.08 (3)
H3A—C3—H3B	108.0	Cl3—Cu1—Cl2	122.51 (3)
N2—C4—C3	109.19 (18)	C1—N1—C3	107.48 (17)
N2—C4—H4A	109.8	C1—N1—C5	108.24 (18)
C3—C4—H4A	109.8	C3—N1—C5	108.54 (18)
N2—C4—H4B	109.8	C1—N1—Cu1	111.19 (14)
C3—C4—H4B	109.8	C3—N1—Cu1	111.95 (13)
H4A—C4—H4B	108.3	C5—N1—Cu1	109.34 (14)
N1—C5—C6	111.69 (18)	C7—N2—C2	111.36 (18)
N1—C5—H5A	109.3	C7—N2—C6	108.14 (18)
C6—C5—H5A	109.3	C2—N2—C6	109.45 (19)
N1—C5—H5B	109.3	C7—N2—C4	111.33 (18)
C6—C5—H5B	109.3	C2—N2—C4	108.25 (18)
H5A—C5—H5B	107.9	C6—N2—C4	108.26 (18)
N2—C6—C5	109.06 (18)	H10—O1—H11	109 (5)
N2—C6—H6A	109.9	Cu1—O2—H12	116 (3)
C5—C6—H6A	109.9	Cu1—O2—H13	113 (2)
N2—C6—H6B	109.9	H12—O2—H13	104 (4)
C5—C6—H6B	109.9		

### Hydrogen-bond geometry (Å, º)

**Table:**

*D*—H···*A*	*D*—H	H···*A*	*D*···*A*	*D*—H···*A*
O2—H13···Cl2^i^	0.93 (4)	2.24 (4)	3.128 (2)	159 (3)
O2—H12···O1	0.80 (4)	1.92 (4)	2.693 (3)	160 (4)
O1—H11···Cl3^ii^	0.77 (5)	2.72 (5)	3.447 (3)	159 (4)
O1—H10···Cl3^i^	0.80 (5)	2.54 (6)	3.337 (3)	171 (5)

Symmetry codes: (i) −*x*+1/2, −*y*+1/2, −*z*+1; (ii) *x*, −*y*, *z*+1/2.
